# Canine Leishmaniosis in Liguria, Northwest Italy: A Nine-Year Retrospective Study in a Historically Endemic Area

**DOI:** 10.3390/ani16111683

**Published:** 2026-05-30

**Authors:** Sara A. Chiarlone, Aitor Garcia-Vozmediano, Luca Carisio, Nicola Pussini, Valentino Revello, Francesca Mancianti, Walter Martelli, Walter Mignone, Monica Dellepiane, Elisabetta Razzuoli, Lisa Guardone

**Affiliations:** 1 S.S. Ponente Ligure, Istituto Zooprofilattico Sperimentale del Piemonte, Liguria e Valle d’Aosta, Via Martini 6, 17056 Savona, Italy; sara.chiarlone@izsplv.it (S.A.C.); nicola.pussini@izsplv.it (N.P.); valentino.revello@izsplv.it (V.R.); waltermignone.wm@gmail.com (W.M.); dellepiane.vet@gmail.com (M.D.); 2 S.C. Epidemiologia, Istituto Zooprofilattico Sperimentale Piemonte Liguria e Valle D’Aosta, Via Bologna 220 c/o Lanificio di Torino, 10154 Torino, Italy; aitor.garciavozmediano@izsplv.it (A.G.-V.); luca.carisio@izsplv.it (L.C.); walter.martelli@izsplv.it (W.M.); 3 Department of Veterinary Sciences, University of Pisa, Viale delle Piagge 2, 56124 Pisa, Italy; francesca.mancianti@unipi.it; 4 S.S. Genova e Portualità Marittima, Istituto Zooprofilattico Sperimentale del Piemonte, Liguria e Valle d’Aosta, Piazza Borgo Pila 39-24, 16129 Genova, Italy; elisabetta.razzuoli@izsplv.it

**Keywords:** *Leishmania infantum*, canine vector-borne diseases, dogs, seroprevalence

## Abstract

Canine leishmaniosis (CanL), caused by *Leishmania infantum*, is an endemic zoonotic disease in the Mediterranean basin, with changing epidemiological patterns. This study aimed to evaluate seroprevalence, assess temporal and seasonal trends, compare owned and shelter dogs, and identify dog-level and environmental factors associated with *L. infantum* exposure in Liguria, a historically endemic area of northwest Italy. A retrospective observational study was conducted using diagnostic data from dogs tested for *L. infantum* in Liguria between 2016 and 2024. Serological results were obtained using an indirect immunofluorescence antibody test (IFAT; cut-off ≥ 1:160). The dataset comprised 75,702 dog-year observations (an observation from the same dog in a different year was considered separately) from 36,679 dogs. Descriptive statistics were used to summarise the study population and factors associated with seropositivity were investigated using generalised estimating equation models with a binomial distribution and logit link function, including demographic and environmental variables. Overall, 10.1% (n = 3693) of dogs tested positive at least once. Inter-annual variability and an increased prevalence during summer and autumn were observed. Shelter dogs had higher odds of seropositivity. Exposure risk increased progressively with age and was slightly higher in males, while minor differences were observed between pure-bred and cross-bred dogs. Environmental factors were also associated with seropositivity, showing a higher prevalence in rural areas and a marked spatial gradient from west to east. This study updates the epidemiology of *L. infantum*, highlighting a non-negligible risk for both dogs and humans and the need for targeted surveillance and control strategies.

## Simple Summary

Canine leishmaniosis (CanL), caused by the parasite *Leishmania infantum*, is a sandflies-transmitted disease widespread in the Mediterranean basin, which can also affect humans. This study analysed data from dogs tested in a veterinary public health laboratory in Liguria, northwest Italy, between 2016 and 2024, to evaluate the prevalence and factors favouring exposure risk. More than 36,000 dogs were analysed, and about 10% tested positive at least once in the study period. The prevalence varied over the years and the season, being higher in summer and autumn, when the insect vectors are more active. Dogs living in shelters had a higher risk of testing positive. Moreover, the exposure risk increased with age and was slightly higher in males, while minor differences were found between pure-bred and cross-bred dogs. Environmental factors also played a role: seropositivity was more common in rural areas and in the western part of the region, decreasing towards the east. This study highlights the need for continuous surveillance and targeted prevention and control strategies, as the risk persists for both dogs and humans.

## 1. Introduction

The term leishmaniasis refers to a group of neglected vector-borne parasitic diseases that are widely distributed across tropical and subtropical regions and the Mediterranean basin. They are enzootic and zoonotic infections caused by protozoa of the genus *Leishmania*. To date, more than 50 species of *Leishmania* have been described worldwide. Approximately 30 species infect mammals and over 20 are known to be able to affect humans [1,2].

Canine leishmaniosis (CanL), caused by *Leishmania infantum*, is largely endemic in South America and through the Mediterranean basin, affecting millions of dogs [3]. *Leishmania infantum* is transmitted by female phlebotomine sandflies belonging to the genus *Phlebotomus* (subgenus *Larroussius*) in the Old World and *Lutzomyia* in the New World [2,4]. Domestic dogs represent the principal reservoir host in urban settings [5,6], although several wild species have also been identified as potential sylvatic reservoirs [7,8,9].

*Leishmania infantum* is also responsible in humans for visceral leishmaniasis (VL), a potentially fatal illness, as well as for cutaneous leishmaniasis (CL). Transmission occurs through the bite of infected sandflies [5]. VL and CL are endemic in all countries of the Mediterranean basin, although the incidence of CanL is considerably higher [5,10]. Nowadays, leishmaniases are still widespread endemic or emerging infections in the EU and its neighbourhood [10]. In Italy human leishmaniasis is a notifiable disease, but underreporting remains a concern [10,11]. Dogs infected with *L*. *infantum* represent a serious threat for both animal and public health [12]. Furthermore, given their high susceptibility, dogs are considered reliable sentinel hosts [13,14].

Southern and coastal areas of Southern European countries such as Italy, France, Greece, Spain and Portugal have historically been recognised as endemic areas [12]. However, recent studies have documented a northward expansion of *L*. *infantum* infection in dogs, now including regions of Northern Italy as well as parts of Northern France and Spain [15,16]. Moreover, imported cases are increasingly described in non-endemic countries such as Germany or the United Kingdom [17,18]. Several factors contribute to this changing epidemiological scenario. Climate change, particularly rising winter temperatures, has facilitated the expansion of phlebotomine vectors towards higher latitudes and altitudes [16,19]. Additionally, increased mobility of dogs across Europe, either through travel with owners or adoption from endemic regions, plays a significant role. In this respect, asymptomatic infected dogs pose a particular risk, as they remain infectious to sandflies [20].

Clinically, CanL is characterised by a wide range of symptoms, from cutaneous lesions and renal dysfunction to systemic disease. Common clinicopathological findings include hyperglobulinemia, hypoalbuminemia, mild to moderate non-regenerative anaemia, and varying degrees of proteinuria. This clinical heterogeneity is influenced by factors such as parasite strain virulence, host immune response, and the presence of co-infections with other vector-borne pathogens [3,21]. Consequently, diagnosis on a specific subject should rely on a comprehensive approach integrating clinical evaluation, medical history, and laboratory findings, including both parasitological and immunological tests to correctly orient therapy [22]. Serology is the preferred method for CanL diagnosis, even during the early stages of the disease, and, among available serological methods, the indirect fluorescence antibody test (IFAT), enzyme-linked immunosorbent assay (ELISA) and rapid immunochromatographic strip tests are widely used [2]. Due to its high sensitivity and specificity in endemic areas, IFAT has long been recommended by the World Organization for Animal Health (WOAH) as the reference serological test [22]; however, it is now regarded as comparable to ELISA [2].

Control strategies for *L*. *infantum* mainly rely on the use of insecticide and repellent compounds, such as pyrethroids, permethrin, and deltamethrin, to prevent sandfly bites. In addition, two vaccines have been introduced in the European Union (EU): CaniLeish^®^ (Virbac, Carros, France) and LetiFend^®^ (LETI Pharma, Barcelona, Spain) [23]. However, CaniLeish^®^ was withdrawn from the EU market in 2023 [24], and currently only LetiFend^®^ remains commercially available. The use of vaccines may pose challenges to the fields of diagnosis, epidemiology and surveillance of the parasite, although LetiFend^®^ does not appear to interfere with common serological tests [23].

The prevalence of CanL varies greatly across time and space, reflecting the influence of environmental, climatic and ecological factors affecting both vector and reservoir dynamics [9,16,25]. A large-scale review in Western Europe reported an overall seroprevalence of 23.2% (median: 10%) based on more than 500,000 dogs from Italy, France, Spain, and Portugal tested between 1971 and 2006 [26]. In Portugal, for instance, a twofold increase in seroprevalence among domestic dogs over a 10-year period was observed [27]. In Italy, CanL is widely distributed, although with a marked geographical heterogeneity [16,28]. CanL was first recorded in Italy at the beginning of the XX century. Historically, endemic areas for human and canine leishmaniosis from the late 1980s to the early 2000s were concentrated along the Tyrrhenian littoral, southern peninsular regions and the islands [13,29]. Indeed, prior to the 1990s, there was no clear evidence of autochthonous *L*. *infantum* transmission north of the Liguria and Emilia-Romagna regions [16]. The prevalence of CanL may also vary according to individual host risk factors, such as increasing age or sex [2,28]. Other important individual risk factors are linked to exposure: dogs living in rural areas or in shelter facilities are generally considered more exposed, although not all studies agree [14,27]. Indeed, the importance of these factors varies among studies, highlighting the context-dependent nature of their effects.

The present study aimed to investigate the epidemiology of CanL in the Liguria region, a historically endemic area in northwestern Italy, through a large-scale retrospective analysis. Existing data for the region are outdated and mainly limited to the western part [30,31,32]. Specifically, the survey estimated seroprevalence, assessed temporal (annual and seasonal) trends, compared owned and shelter dog populations and evaluated dog-level and environmental risk factors with the additional aim of describing the spatial distribution of seropositivity, while also contributing to public health risk assessment.

## 2. Materials and Methods

### 2.1. Study Area

The study was conducted in Liguria, a mountainous and hilly region of northwest Italy bordering the Ligurian Sea. The region covers an area of 5416 km^2^, and has a population of approximately 1,510,143 inhabitants, corresponding to a density of about 280 inhabitants/km^2^ [33], and density strongly increases in summer due to national and international tourism, mainly from North Italy and countries of Northern Europe (such as Germany, The Netherland, Belgium, and Norway). Liguria is administratively divided into four provinces (Imperia, Savona, Genova and La Spezia) and is characterised by a narrow coastal strip, with a maximum inland extension of approximately 35 km. The Ligurian Sea exerts a strong moderating influence on the climate, while the mountain range acts as a barrier against cold northern winds. Climatic conditions vary across the region. The western province (Imperia) is characterised by particularly mild temperatures (average: 9 °C in January and 25 °C in July), whereas slightly lower winter temperatures are observed in the central provinces (Savona and Genoa: around 7 °C in January). Precipitation is more abundant in Genoa and in the eastern part of the region (1000–1500 mm/year). Additional variability occurs between coastal and inland areas, as well as between slopes facing the sea and those oriented northwards, where colder temperatures (down to 0 °C in January) and higher rainfall (>2000 mm/year) are recorded [34].

### 2.2. Ethical Statement

All data included in this retrospective study were obtained from the diagnostic records of the Istituto Zooprofilattico Sperimentale del Piemonte, Liguria e Valle d’Aosta (IZSPLV). Samples were collected within routine veterinary clinical activity and health controls conducted by the Public Veterinary Service. No additional sampling or experimental procedures were performed specifically for this study.

### 2.3. Study Design and Population

A retrospective observational study was conducted using repeated dog-year observations (an observation from the same dog in a different year was considered separately) from dogs residing in the Liguria region and tested for *L*. *infantum* between 2016 and 2024. Data were retrieved from diagnostic records generated by IZSPLV. Analysed samples originated from dogs across the region, including both shelter dogs and privately owned animals examined by veterinary practitioners. Shelter dogs were tested either upon admission to kennels (Savona, Genova and La Spezia provinces) and/or during annual preventive screening programmes (Imperia). Owned dogs were tested within routine veterinary practice, including preventive screening, post-adoption evaluations, clinical suspicion or follow up after treatment. Each record corresponded to a serological test performed on an individual dog in a given year. Only dogs uniquely identified by a microchip number were included, allowing repeated observations of the same dog to be linked over time. The final analytical dataset was structured at the dog-year level, whereby a single dog could contribute multiple observations across the study period.

### 2.4. Laboratory Analysis: Indirect Fluorescence Antibody Test (IFAT)

Serological analyses were performed at the IZSPLV laboratories in Savona and Imperia. Upon arrival, each sample was assigned a unique identification code linked to the municipality of origin, the microchip number and the housing type (private household/kennel). Samples were stored at +4 °C and analysed within 3 days. Serum samples were tested for IgG antibodies against *L*. *infantum* antigen using IFAT slides containing fixed promastigotes. Initially, sera were screened by a serial two-fold dilutions up to a 1:40 ratio in phosphate-buffered saline (PBS, pH 7.2) followed by incubation for 30 min at 37 °C in a humidified chamber. Positive and negative canine control sera, provided by the National Reference Centre for Leishmaniosis—CRENAL (Istituto Zooprofilattico Sperimentale della Sicilia) were included on each slide. Then, the IFAT slides were rinsed in PBS twice and in distilled water once and air-dried. Subsequently, slides were incubated with fluorescein isothiocyanate—conjugated (FITC) anti-canine IgG, also supplied by CRENAL, for 30 min at 37 °C. Slides were then washed, air-dried and examined under fluorescence microscope (NE620, Nexcope, Istanbul, Turkey). Samples showing fluorescence at the 1:40 dilution were further titrated by serial two-fold dilutions (1:40 to 1:5120). A titre of 1:160 was considered positive, in accordance with the national reference centre—CRENAL [35] and WOAH [2].

### 2.5. Data Management and Processing

A set of individual and territorial variables potentially associated with exposure risk was considered. Dogs were classified according to their origin as either owned dogs (privately owned animals presented to veterinary clinics) or shelter dogs housed in municipal kennels. This classification was used to capture potential differences in management conditions, exposure patterns and health monitoring between the two populations. Individual dog characteristics included sex, age and breed, retrieved from the Italian Pet Identification System (Sistema informativo Nazionale degli Animali da Compagnia; https://www.vetinfo.it/). Age was categorised in four classes: <1 year, 1–3 years, 4–6 years and >6 years. Breed was classified as pure-bred and cross-bred.

To account for spatial variability, the province of residence (i.e., Imperia, Savona, Genoa, and La Spezia) was included as a geographical variable. In addition, the degree of urbanisation of the municipality of residence was used as a proxy for environmental conditions influencing vector presence and transmission dynamics. Municipalities were classified as rural areas, small towns or suburban areas and large cities, according to the Italian National Institute of Statistics [33]. More detailed environmental and climatic covariates were not integrated into the present analysis, as the study was primarily designed as a retrospective surveillance-based investigation using routinely collected diagnostic data rather than a dedicated ecological modelling approach.

Breed was explored descriptively but was not included in the final multivariable model, as it was considered a proxy for exposure-related factors already captured by dog population, age, and urbanisation level. Records with missing information for individual-level variables were retained in the analyses and categorised as “Unknown” to avoid substantial loss of observations associated with complete-case exclusion. Given the surveillance-based nature of the dataset, missing information may also reflect differences in management or testing contexts and was therefore modelled explicitly rather than omitted.

### 2.6. Statistical Analyses

Descriptive statistics were used to summarise the characteristics of the study population. For descriptive purposes at the individual level, dogs were classified as positive if at least one positive serological test was recorded during the study period. As mentioned, (Section 2.4), serological positivity was defined using a cut-off titre of ≥1:160, otherwise dogs were considered as negative in the statistical analyses. For each covariate category, the number and percentage of dogs were calculated separately for owned and shelter populations, as well as for the overall sample. Seroprevalence was estimated together with 95% confidence intervals (95% CIs) using the exact binomial method. Temporal patterns of seropositivity were explored using dog-year observations to estimate annual seroprevalence. In addition, monthly seroprevalence was calculated and summarised using a three-month moving average to reduce short-term variability due to fluctuations in testing volume.

Factors associated with *Leishmania* seropositivity were investigated using generalised estimating equation models with a binomial distribution and logit link function. This approach provides population-averaged estimates while accounting for the correlation between repeated observations from the same individual. Clustering was defined at the dog level using the microchip identification code. An exchangeable correlation structure was specified, assuming a constant correlation between repeated observations within the same dog.

The outcome variable was the serological status (positive vs. negative) at the dog-year level. The explanatory variables included the year of observation, dog population (owned vs. shelter), age class, sex, degree of urbanisation and province of residence. Model results were expressed as odds ratios (ORs) with corresponding 95% CIs.

A sensitivity analysis was performed to evaluate the robustness of the results to the diagnostic threshold used to define serological positivity. In this analysis, dogs were considered positive when antibody titres were ≥1:80, corresponding to a more permissive threshold frequently used in the available literature [28,36]. All descriptive and multivariate analyses were repeated using this alternative outcome definition (Supplementary Tables S1 and S2). Results were compared with those obtained in the primary analysis based on the 1:160 cut-off, enabling evaluation of the consistency of the findings and facilitating comparison with previous studies. All statistical analyses and graphical representations were performed using R software (version 4.3.3), while spatial visualisations were generated using QGIS software (version 3.44.8). Statistical significance was assessed using a two-sided α level of 0.05.

## 3. Results

A total of 36,679 dogs were included in the study, contributing 75,702 dog-year observations between 2016 and 2024. Owned dogs represented the majority of the study population (n = 32,998; 90%), whereas 3681 dogs (10%) originated from shelters (Table 1). Demographic information was partially incomplete: sex and breed were recorded for approximately two-thirds of individuals (67.6%), while age information was available for 60.5% of dogs. Most animals lived in small towns or suburban areas (n = 25,514; 69.6%), followed by rural settings (n = 6135; 16.7%) and large urban centres (n = 5030; 13.7%).

**Figure 1:**
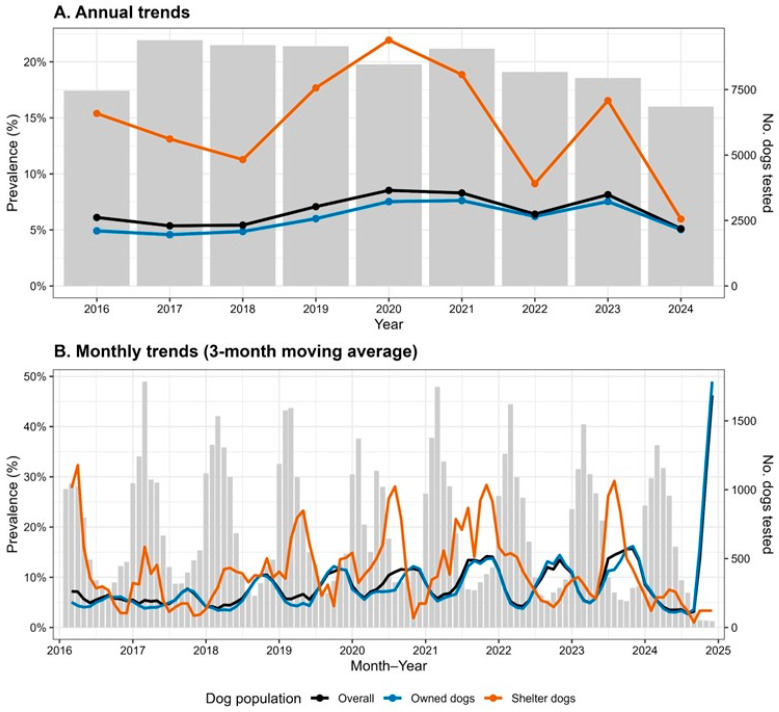
Temporal dynamics of *Leishmania* seropositivity among tested dogs in Liguria, Italy (2016–2024): (**A**) Annual apparent seroprevalence stratified by dog population (owned dogs, shelter dogs, and overall). Lines represent yearly prevalence estimates calculated using dog-year observations, while grey bars indicate the number of dogs tested each year. (**B**) Monthly apparent seroprevalence stratified by dog population, displayed as a three-month moving average to highlight temporal patterns. Grey bars indicate the number of dogs tested each month.

Overall, 3693 dogs tested positive at least once during the study period, corresponding to an individual-level prevalence of 10.1% (95% CI: 9.8–10.4). When *L. infantum* exposure was evaluated at the dog-year level, marked temporal fluctuations emerged (Figure 1). Annual apparent prevalence remained relatively stable between 2016 and 2018 (range: 5.4–6.1%), increased substantially between 2019 and 2021 (7.1–8.5%), reaching a peak in 2020 (8.5%) and 2021 (8.3%), and subsequently declined in 2022 (6.4%). A moderate increase was observed again in 2023 (8.1%), followed by a further decrease in 2024 (5.1%). These temporal patterns were consistent with the results of the adjusted analysis (Table 2), which showed a higher odds of seropositivity in 2020 (OR = 1.24; 95% CI = 1.09–1.40) and 2021 (OR = 1.25; 95% CI = 1.11–1.42) compared with 2016, and a significantly lower risk in 2024 (OR = 0.73; 95% CI = 0.63–0.84). Monthly prevalence estimates revealed a recurrent seasonal pattern, with higher values consistently observed during summer and autumn months (from 7.6% in June up to 11.8% in October; Figure 1). The lowest prevalence values were registered across the first months of the year, with slight variations being recorded between January and May (5.4–5.6%). Short-term variations observed during the final months of 2024 coincided with a marked reduction in the number of tested dogs and were therefore interpreted cautiously, as they likely reflect sampling variability rather than a true epidemiological increase.

Substantial differences were observed between the two dog populations (Figure 1). Shelter dogs showed a markedly higher seroprevalence (14.5%; 95% CI = 13.6–15.4) compared with owned dogs (6.1%; 95% CI = 5.9–6.2). After adjustment for temporal, demographic and territorial factors, this difference remained pronounced (Table 2): shelter dogs had approximately twice the odds of exposure compared with owned dogs (OR = 2.02; 95% CI = 1.86–2.21).

Dog characteristics were also strongly associated with exposure risk. A clear age-related gradient was evident, with seroprevalence increasing progressively with age. Dogs younger than one year consistently showed the lowest prevalence (5.5%; 95% CI = 4.7–6.3) whereas older animals exhibited higher seroprevalence levels, ranging from 8.4% and 13.7% (Table 1). In particular, dogs aged 1–3 years had about twice the odds of exposure (OR = 2.04; 95% CI = 1.61–2.58) compared with puppies, while the odds increased further among dogs aged 4–6 years (OR = 3.43; 95% CI = 2.72–4.32) and >6 years (OR = 3.93; 95% CI = 3.13–4.93).

Sex-related differences were smaller but consistent. Across most study years, male dogs showed a slightly higher prevalence than females (Supplementary Figure S1), and this pattern persisted after adjustment, with males showing 25% higher odds of exposure (OR = 1.25; 95% CI = 1.16–1.34). Descriptive analyses of breed revealed only minor differences in seroprevalence between pure-bred dogs (9.8%; 95% CI = 9.4–10.3) and cross-bred dogs (10.5%; 95% CI = 9.9–11.1), while dogs with unknown breed showed an intermediate prevalence (10.0%; 95% CI = 9.5–10.6; Table 1). These findings suggest that breed alone did not appear to substantially influence exposure risk in this population. Dogs with unknown sex and age also exhibited somewhat higher odds of seropositivity (Table 2), suggesting that these individuals may belong to higher-risk populations.

**Figure 2:**
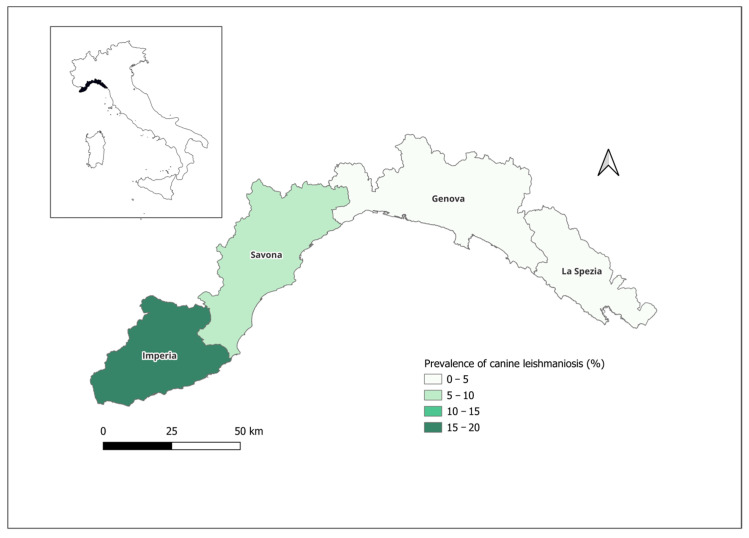
Geographical pattern of *Leishmania infantum* prevalence in dogs in the four provinces of Liguria, Italy (2016–2024).

Environmental context also influenced the exposure risk. The seroprevalence of *L*. *infantum* was generally higher in rural environments (11.7%; 95% CI = 10.9–12.5) and decreased with increasing levels of urbanisation (Table 1). Indeed, compared with dogs living in rural areas, those residing in small towns or suburban settings and large urban centres displayed lower risk levels, with odds of seropositivity of 0.76 (95% CI = 0.71–0.82) and 0.56 (95% CI = 0.48–0.65), respectively. Also, we observed a marked spatial heterogeneity in seropositivity within the study area, which followed a west-to-east decreasing gradient (Figure 2). Dogs residing in Imperia province showed the highest seroprevalence levels (16.6%; 95% CI = 15.9–17.3), with respect to dogs residing in the remaining provinces (Table 1). Accordingly, the odds of seropositivity decreased gradually in dogs residing in Savona, Genova and La Spezia provinces (Table 2).

Sensitivity analyses based on the alternative serological threshold (titre ≥ 1:80) yielded results that are highly consistent with those of the primary analysis using the ≥1:160 cut-off, including the elevated odds of seropositivity observed among shelter dogs and the progressive increase in seropositivity with age (Supplementary Tables S1 and S2). Likewise, complete-case analyses excluding observations with missing demographic information retained 47,891 dog-year observations (63.3% of the original dataset) and yielded comparable estimates across all examined covariates (Supplementary Table S3).

## 4. Discussion

Canine leishmaniosis remains an important zoonotic infection in the Mediterranean basin, with ongoing transmission in several historically and newly endemic areas [12,13,14,15,16]. In this large retrospective study, we found an overall seroprevalence of 10.1% against *L*. *infantum* in dogs in Liguria, confirming the persistence of endemic transmission in the region. Infection patterns were characterised by marked temporal variability without a clear long-term trend, a consistent seasonal peak during summer–autumn months, and substantial heterogeneity between dog populations and geographical areas.

The prevalence observed in this study is consistent with the endemic nature of CanL in Mediterranean regions and falls within the range reported in previous studies conducted in southern Europe [26,37]. However, comparisons across studies should be interpreted with caution due to differences in study design, sampled populations, and diagnostic protocols. In particular, seroprevalence estimates may vary according to the IFAT cut-off adopted, which is not standardised across epidemiological surveys, ranging from ≥1:40 to ≥1.160 [28,29,30,35,36,38]. In our study, sensitivity analyses performed using a lower serological threshold (≥1:80) yielded results that are highly consistent with those obtained using the ≥1:160 cut-off, both in terms of prevalence estimates and associations with the examined covariates. This supports the robustness of our findings and indicates that the main epidemiological patterns identified are not substantially influenced by the choice of diagnostic threshold. Nevertheless, IFAT detects anti-*Leishmania* antibodies and therefore a single serological test could only indicate exposure to *L*. *infantum* rather than confirmed active infection, especially in the absence of data on the clinical status. Consequently, seropositive dogs may include asymptomatic, previously exposed or clinically infected animals, and unfortunately no data on parasitological or molecular confirmation was available. Moreover, IFAT interpretation may be further limited by cross-reactivity and individual variability in antibody response [2,23].

No clear increasing or decreasing trend was observed over the study period; rather, annual fluctuations were evident, with higher prevalence values recorded between 2019 and 2021. This pattern is consistent with a stable endemic context, in which transmission persists over time without marked long-term expansion or decline. However, temporal variability may reflect not only ecological fluctuations affecting vector abundance and transmission dynamics, but also changes in surveillance intensity, testing practices and composition of the tested population across years. In particular, the higher prevalence observed during 2020–2021 should be interpreted cautiously. Although a true increase in transmission cannot be excluded, the COVID-19 pandemic may also have indirectly influenced veterinary healthcare access, testing behaviour, and sampling patterns, potentially affecting the composition of the tested dog population. Unfortunately, detailed information regarding testing indications and surveillance intensity was not systematically available, preventing a more formal assessment of these effects. Similarly, the decline observed in 2024 coincided with a reduction in the number of tested dogs, suggesting that part of the observed variation may reflect sampling fluctuations rather than true epidemiological changes.

Although the long-standing awareness of CanL in the region may have contributed to the adoption of preventive measures, the absence of reliable data on vaccination coverage and use of repellents does not allow a direct assessment of their impact on infection trends. Overall, the absence of a sustained long-term increase or decline is compatible with a stable endemic transmission pattern in the study area. Different temporal patterns have been reported in other settings. For instance, a slight decrease in prevalence was observed in Sicily between 2020 (25.4%) and 2021 (21.6%) [35], whereas an increasing trend was reported in Portugal, where prevalence doubled over a 10-year period [27]. These discrepancies highlight the influence of local ecological, epidemiological, and management factors on transmission dynamics.

A clear seasonal pattern was identified, with higher prevalence during summer and autumn months, declining in the winter. This pattern has been described before [39] and is biologically plausible, as phlebotomine sandfly activity typically begins in late spring [40], while seroconversion occurs within a few months following infection [41]. Conversely, lower prevalence values observed during winter and early spring likely reflect reduced recent transmission and support current recommendations to perform screening during this period, when exposure-related bias is minimised.

A marked difference in seroprevalence was observed between dog populations, with shelter dogs showing significantly higher values compared with owned animals (14.5% vs 6.1%) and approximately twice the odds of seropositivity after adjustment (OR = 2.02). This finding is consistent with previous studies reporting higher seroprevalence against *L*. *infantum* in shelter dog populations [42], and may reflect a combination of factors, including previous exposure before admission, heterogeneous origin and less consistent use of preventive measures (e.g., repellents). However, contrasting results have been reported in the literature, with some studies showing a similar or even lower prevalence in shelter dogs compared to owned animals [14,43,44,45], and others not identifying kennel status as a risk factor [38]. These discrepancies likely reflect differences in shelter management practices (e.g., animal testing and prevention), as well as variability in study design and sampling strategies. In particular, variability in testing indications, including routine screening, clinical suspicion and post-treatment follow-up, may introduce selection bias in diagnostic datasets. Previous studies have highlighted that owned dogs are often tested under clinical suspicion or follow up, potentially leading to an overrepresentation of infected animals [28]. In our study, detailed information regarding the reason for testing was not systematically available, preventing stratified analyses according to testing indication and limiting the possibility of directly quantifying the impact of this bias. Furthermore, owned dogs represented the majority of the study population, which may have influenced overall prevalence estimates. Although the multivariate analysis adjusted for major demographic and geographical covariates, residual bias related to testing indications and differential healthcare-seeking behaviours cannot be excluded. Therefore, the observed association between dog population and infection risk should be interpreted with caution, as they may partly reflect differences in prior exposure history and testing practices between the two dog populations.

Age was strongly associated with exposure risk, with seroprevalence and odds of seropositivity increasing progressively with age. Dogs younger than one year showed the lowest prevalence (5.5%), whereas older animals exhibited higher seroprevalence levels, supporting the role of cumulative exposure in endemic settings. This pattern is consistent with several studies reporting a higher seroprevalence in adult and older dogs [27,28,44,45,46,47]. Although alternative age distributions, including bimodal patterns, have been described in some settings [3,48,49], our findings support a predominantly progressive increase in exposure risk with age, consistent with stable endemic transmission.

Sex-related differences were less pronounced, although male dogs showed a moderately higher risk of seropositivity compared with females. This finding agrees with studies reporting higher seroprevalence values in males [28,30,38,44] and is generally attributed to behavioural factors or common human habits, such as increased outdoor exposure, especially in rural settings, or the use of males as guard animals. However, evidence in the literature remains inconsistent [27,28,47], suggesting that sex is likely a secondary risk factor compared with other variables.

No substantial differences were observed between pure-bred and cross-bred dogs, in agreement with studies reporting no clear association between breed and infection risk [27,47]. Although some evidence suggests breed-related differences in susceptibility, potentially linked to genetic or immunological factors [3,28], these effects appear to be context-dependent and were not evident in our study population. Unfortunately, data on the use of repellents, vaccination status, travel history, or clinical condition were not included in the analysed database, nor are they comprehensively available in the Italian Pet Identification System. Indeed, although we acknowledge that missing the inclusion of such data is a limitation of the study, the selected variables were the only robust data available. Other similar large studies [28] experienced analogous limitations.

In addition to individual characteristics, environmental and ecological factors play a crucial role in shaping the epidemiology of *L*. *infantum* [50]. In Mediterranean regions, climatic conditions characterised by mild winters, and warm summers favour the survival and seasonal activity of phlebotomine sandflies [40], supporting endemic transmission. In Liguria, the combination of coastal, peri-urban and inland rural environments within a limited geographic area likely contributes to the ecological suitability for vector persistence. In addition, climate change has been associated with the expansion of vector distribution toward new areas, including previously marginal or non-endemic regions [19]. The presence of competent vector species in the study area, such as *Phlebotomus perniciosus*, *P*. *ariasi* and *Sergentomyia minuta* [51], further supports the potential for sustained transmission. Data on potential wildlife reservoirs, which are known to be able to play a role in some areas, are available for foxes from Imperia, where 18% of the 50 animals examined presented antibodies against *Leishmania* spp. [7]. However, although environmental and climatic variables could potentially contribute to explaining local transmission dynamics, their integration was beyond the scope of the present surveillance-based analysis, which primarily relied on routinely collected diagnostic data. Instead, province of residence and degree of urbanisation were included as proxy indicators of ecological and territorial heterogeneity potentially associated with vector distribution and exposure risk. Future studies integrating entomological, climatic and environmental data through dedicated spatial and ecological approaches would be valuable to better characterise local transmission dynamics and environmental drivers of seropositivity.

In the present study, seroprevalence was higher in dogs living in rural environments and decreased with increasing levels of urbanisation. This pattern likely reflects more suitable ecological conditions for sandfly vectors in rural settings, as well as increased outdoor exposure of dogs, particularly those used for activities such as hunting. Similar findings have been reported in other endemic areas, including Portugal [45], and in a global systematic review [14]. However, high seroprevalence has also been described in peri-urban areas of large European cities, where specific environmental conditions may still support vector presence [52], suggesting that transmission risk is influenced by local ecological and behavioural factors rather than by urbanisation alone.

The marked west-to-east gradient observed in our study suggests substantial spatial heterogeneity in the distribution of *L*. *infantum* within Liguria, with the highest prevalence values recorded in Imperia province. This finding is consistent with historical data documenting long-standing endemic transmission in western Liguria since at least the early 1990s (15–30.3%) [7,30]. In contrast, lower prevalence values were observed in the eastern provinces, for which published epidemiological data remain limited. In addition, differences in diagnostic coverage and reporting pathways across the region may also have influenced the observed geographical distribution, particularly in eastern areas where a proportion of diagnostic activity may occur outside the institutional surveillance network considered in the present study. Since the spatial analyses performed were descriptive in nature, the overall geographical patterns should be interpreted cautiously.

## 5. Conclusions

From a public health perspective, these findings are particularly relevant in the context of limited and heterogeneous surveillance systems. In Italy, CanL has not been subjected to a nationally harmonised monitoring strategy, and current European regulations do not include it among diseases requiring harmonised surveillance. Although recent national guidelines have promoted a One Health approach for the control of leishmaniasis [11], implementation remains largely regional. Given the higher-than-national average incidence of human leishmaniasis in Liguria (1.59 vs. 0.70) [50], together with the persistent endemicity observed in the investigated canine population and the high touristic vocation, the development of a regional integrated surveillance plan, combining veterinary, human and entomological data, should be considered.

## Figures and Tables

**Table 1:** Characteristics of the study population and seroprevalence of *Leishmania* among owned and shelter dogs in Liguria, Italy (2016–2024).

	Owned Dogs (n = 33,009)		Shelter Dogs (n = 3670)		Total	
	No. of Individuals (%)	Seroprevalence (%) [95% CI]	No. of Individuals (%)	Seroprevalence (%) [95% CI]	No. of Individuals (%)	Seroprevalence (%) [95% CI]
**Sex**						
Female	11,085 (33.6)	8.3 [7.8–8.8]	993 (27)	14.8 [12.7–17.2]	12,078 (32.9%)	8.8 [8.3–9.3]
Male	11,185 (33.9)	10.5 [9.9–11.1]	1547 (42)	17.0 [15.2–19.0]	12,732 (34.7%)	11.3 [10.7–11.8]
Unknown	10,728 (32.5)	9.1 [8.6–9.7]	1141 (31)	19.0 [16.8–21.4]	11,869 (32.4%)	10.1 [9.5–10.6]
**Age class (in years)**						
<1y	2915 (8.8)	4.7 [4.0–5.5]	458 (12.4)	10.5 [7.8–13.7]	3373 (9.2%)	5.5 [4.7–6.3]
1–3y	8443 (25.6)	7.6 [7.0–8.1]	1005 (27.3)	15.8 [13.6–18.2]	5285 (14.4%)	8.4 [7.9–9.0]
4–6y	3709 (11.2)	11.7 [10.7–12.8]	369 (10)	17.9 [14.1–22.2]	9448 (25.8%)	12.3 [11.3–13.3]
>6y	4843 (14.7)	13.2 [12.2–14.2]	442 (12)	19.2 [15.7–23.2]	4078 (11.1%)	13.7 [12.8–14.6]
Unknown	13,088 (39.7)	9.3 [8.8–9.8]	1407 (38.2)	19.1 [17.1–21.3]	14,495 (39.5%)	10.3 [9.8–10.8]
**Breed**						
Cross-bred	8003 (24.3)	9.2 [8.6–9.9]	1915 (52)	15.7 [14.1–17.4]	9918 (27.0%)	10.5 [9.9–11.1]
Pure-bred	14,231 (43.1)	9.5 [9.0–10.0]	623 (16.9)	17.7 [14.7–20.9]	14,854 (40.5%)	9.8 [9.4–10.3]
Unknown	10,764 (32.6)	9.1 [8.5–9.6]	1143 (31.1)	19.0 [16.7–21.4]	11,907 (32.5%)	10.0 [9.5–10.6]
**Province**						
Imperia (W)	9977 (30.2)	14.0 [13.3–14.6]	1798 (48.8)	31.3 [29.2–33.5]	11,775 (32.1)	16.6 [15.9–17.3]
Savona (NW)	20,341 (61.6)	7.6 [7.2–8.0]	533 (14.5)	5.8 [4.0–8.2]	20,874 (56.9)	7.5 [7.2–7.9]
Genova (NE)	1546 (4.7)	3.8 [2.9–4.9]	586 (15.9)	2.9 [1.7–4.6]	2132 (5.8)	3.6 [2.8–4.4]
La Spezia (E)	1134 (3.4)	6.3 [4.9–7.8]	764 (20.8)	2.1 [1.2–3.4]	1898 (5.2)	4.6 [3.7–5.6]
**Urban level**						
Rural areas	5876 (17.8%)	11.6% [10.8–12.5]	262 (7.1%)	13.7% [9.8–18.5]	6138 (16.7%)	11.7% [10.9–12.5]
Small towns or suburban areas	22,959 (69.6%)	9.5% [9.1–9.9]	2555 (69.4%)	22.4% [20.8–24.1]	25,514 (69.6%)	10.8% [10.4–11.1]
Large cities	4163 (12.6%)	5.0% [4.4–5.7]	864 (23.5%)	2.2% [1.3–3.4]	5027 (13.7%)	4.5% [4.0–5.1]

Note: Seroprevalence estimates are calculated at the individual level, classifying dogs as positive if at least one positive test (≥1:160) was recorded during the study period.

**Table 2:** Factors associated with *Leishmania infantum* seropositivity among dogs in Liguria, northwestern Italy (2016–2024).

Variable	Category	Odds Ratio (OR) [95% CI]	*p*-Value
Year	2016	1.00 (ref.)	
	2017	0.79 [0.69–0.90]	<0.001
	2018	0.78 [0.68–0.89]	<0.001
	2019	1.02 [0.90–1.15]	0.810
	2020	1.24 [1.09–1.40]	<0.001
	2021	1.25 [1.11–1.42]	<0.001
	2022	0.96 [0.84–1.10]	0.593
	2023	1.27 [1.12–1.44]	<0.001
	2024	0.73 [0.63–0.84]	<0.001
Dog population	Owned dogs	1.00 (ref.)	
	Shelter dogs	2.02 [1.86–2.21]	<0.001
Age class (in years)	<1	1.00 (ref.)	
	1–3	2.04 [1.61–2.58]	<0.001
	4–6	3.43 [2.72–4.32]	<0.001
	>6	3.93 [3.13–4.93]	<0.001
	Unknown	2.94 [2.31–3.75]	<0.001
Sex	Female	1.00 (ref.)	
	Male	1.25 [1.16–1.34]	<0.001
	Unknown	1.33 [1.18–1.51]	<0.001
Urban level	Rural	1.00 (ref.)	
	Small town or suburban areas	0.76 [0.71–0.82]	<0.001
	Large cities	0.56 [0.48–0.65]	<0.001
Province	Imperia	1.00 (ref.)	
	Genova	0.31 [0.24–0.39]	<0.001
	La Spezia	0.45 [0.37–0.55]	<0.001
	Savona	0.42 [0.40–0.45]	<0.001

Note: Reference categories (ref.) are indicated for each variable (OR = 1.00). Odds ratios greater than 1 indicate increased odds of seropositivity relative to the reference category.

## Data Availability

The original contributions presented in this study are included in the article/Supplementary Materials. Further inquiries can be directed to the corresponding author.

## Supplementary Materials

The following supporting information can be downloaded at: https://www.mdpi.com/article/10.3390/ani16111683/s1, Figure S1. Annual apparent prevalence of Leishmania infection among tested dogs in Liguria, Italy, stratified by sex (2016–2024); Table S1. Characteristics of the study population and prevalence of *Leishmania* infection among owned and shelter dogs in Liguria, Italy (2016–2024), using the alternative serological positivity threshold (antibody titre ≥ 1:80); Table S2. Output of the sensitivity analysis based on the alternative serological positivity threshold (antibody titre ≥ 1:80); Table S3. Output of the sensitivity analysis excluding dog-year observations with missing information for age or sex.

## Abbreviations

The following abbreviations are used in this manuscript:

CanL	Canine leishmaniosis
CL	Cutaneous leishmaniasis
CRENAL	National Reference Centre for Leishmaniosis
ELISA	Enzyme-linked immunosorbent assay
EU	European Union
IFAT	Indirect fluorescence antibody test
FICT	Fluorescein isothiocyanate—conjugated
PBS	Phosphate-buffered saline
VL	Visceral leishmaniasis
