# Supplementary figures and images for: Canine Leishmaniosis in Liguria, Northwest Italy: A Nine-Year Retrospective Study in a Historically Endemic Area
Source: Animals (Basel). 2026 May 30;16(11):1683. doi: 10.3390/ani16111683 (PMC13255584; doi:10.3390/ani16111683)

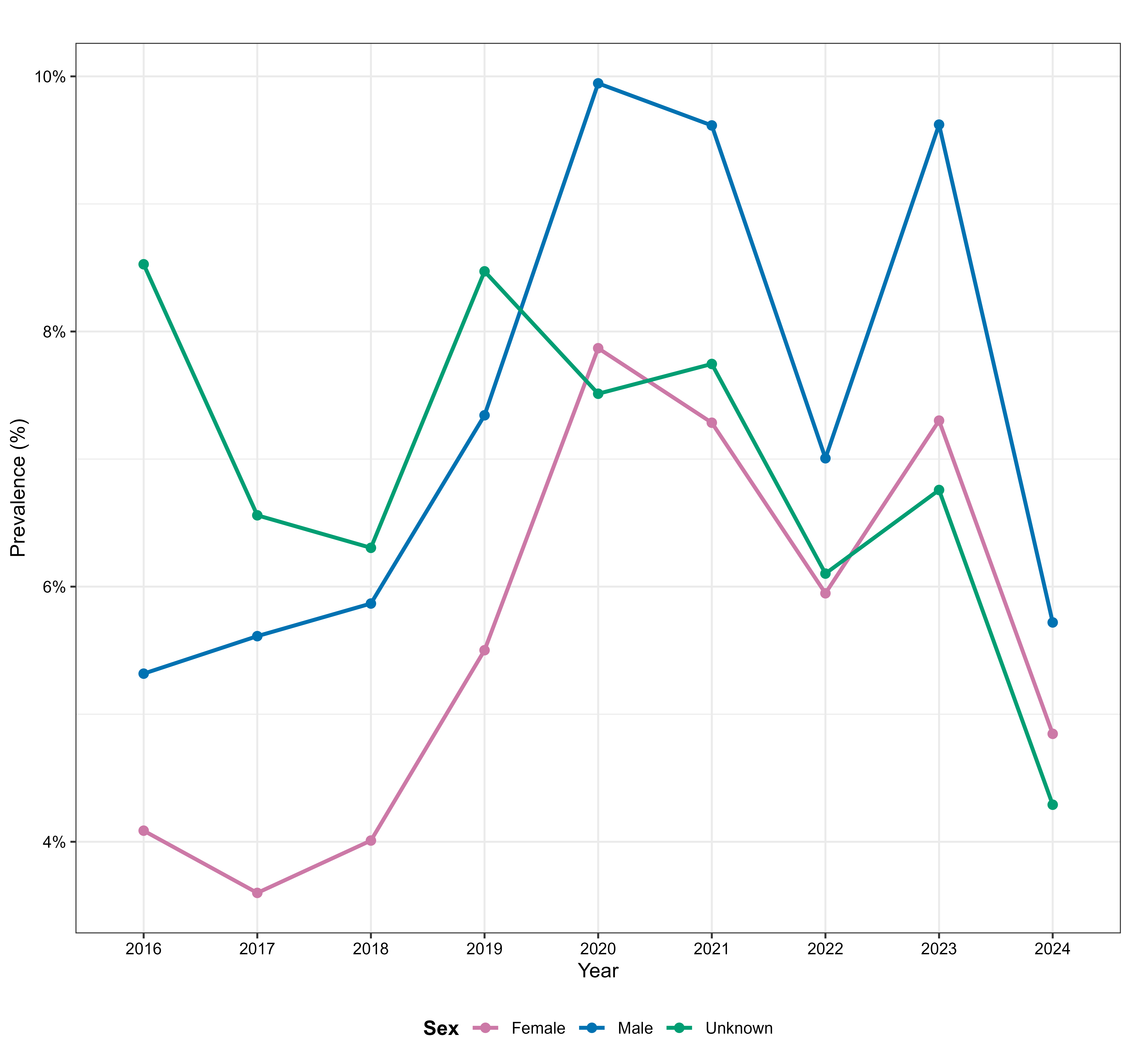

Supplement: Supplementary file 1 [file animals-16-01683-s001.zip › animals-4289840-supplementary.png]
